# The effect of COVID-19 vaccination status on all-cause mortality in patients hospitalised with COVID-19 in Hungary during the delta wave of the pandemic

**DOI:** 10.1007/s11357-023-00931-1

**Published:** 2023-09-27

**Authors:** Viktor J. Horvath, Magdolna Békeffy, Zsuzsanna Németh, Emese Szelke, Vince Fazekas-Pongor, Noémi Hajdu, Márk M. Svébis, József Pintér, Beatrix A. Domján, Szilvia Mészáros, Anna E. Körei, Árpád Kézdi, Ibolya Kocsis, Katalin Kristóf, Péter Kempler, Ferenc Rozgonyi, István Takács, Adam G. Tabák

**Affiliations:** 1 Department of Internal Medicine and Oncology, Semmelweis University Faculty of Medicine, 2/a Korányi S. Str, 1083 Budapest, Hungary; 2 Department of Public Health, Semmelweis University Faculty of Medicine, Budapest, Hungary; 3 Department of Laboratory Medicine, Semmelweis University Faculty of Medicine, Budapest, Hungary; 4 UCL Brain Sciences, University College London, London, UK

**Keywords:** COVID-19 disease, Hospitalisation, Vaccination, Mortality

## Abstract

The high mortality of patients with coronavirus disease 2019 (COVID-19) is effectively reduced by vaccination. However, the effect of vaccination on mortality among hospitalised patients is under-researched. Thus, we investigated the effect of a full primary or an additional booster vaccination on in-hospital mortality among patients hospitalised with COVID-19 during the delta wave of the pandemic. This retrospective cohort included all patients (*n* = 430) admitted with COVID-19 at Semmelweis University Department of Medicine and Oncology in 01/OCT/2021–15/DEC/2021. Logistic regression models were built with COVID-19-associated in-hospital/30 day-mortality as outcome with hierarchical entry of predictors of vaccination, vaccination status, measures of disease severity, and chronic comorbidities. Deceased COVID-19 patients were older and presented more frequently with cardiac complications, chronic kidney disease, and active malignancy, as well as higher levels of inflammatory markers, serum creatinine, and lower albumin compared to surviving patients (all* p* < 0.05). However, the rates of vaccination were similar (52–55%) in both groups. Based on the fully adjusted model, there was a linear decrease of mortality from no/incomplete vaccination (ref) through full primary (OR 0.69, 95% CI: 0.39–1.23) to booster vaccination (OR 0.31, 95% CI 0.13–0.72, *p* = 0.006). Although unadjusted mortality was similar among vaccinated and unvaccinated patients, this was explained by differences in comorbidities and disease severity. In adjusted models, a full primary and especially a booster vaccination improved survival of patients hospitalised with COVID-19 during the delta wave of the pandemic. Our findings may improve the quality of patient provider discussions at the time of admission.

**Supplementary Information:**

The online version contains supplementary material available at 10.1007/s11357-023-00931-1.

## Introduction

The pandemic of coronavirus disease 2019 (COVID-19) caused by severe acute respiratory syndrome coronavirus-2 (SARS-CoV-2) is still an ongoing problem. Although different vaccinations have proven their excellent efficacy in phase III trials [1–3], the generalisation of the results of randomised clinical trials is limited due to self-selection of participants, limited inclusion of certain population segments, the evolution of the virus itself over time, insufficient power to investigate rare complications, and inability to take into account specific local circumstances.

Obviously, these remaining uncertainties highlight the importance of real-world observational studies. According to a meta-analysis of 51 observational studies, vaccine effectiveness against infection, severe infection, and death in the general population was 86.1%, 89.1%, and 99.0%, respectively. [4] However, vaccine efficacy or effectiveness decreased by 10% against severe and by 25% against symptomatic COVID-19 over 6 months but still mostly remained over 70% against severe disease according to a meta-analysis including both efficacy trials and non-randomised investigations [5]. These observations were largely confirmed by the HUN-VE 3 Study for the delta wave, as well as for the delta and omicron waves in the HUN-VE 2 study for the Hungarian general population. Furthermore, these observational studies provided evidence on the lower vaccine effectiveness of the Janssen and Sinopharm vaccines widely used in Hungary[6, 7].

While the above observations are extremely important for COVID-19-related healthcare planning and for briefing the general public on the benefits of vaccination, they are unable to answer a clinically relevant question whether patients hospitalised with COVID-19 have different mortality and other outcomes by vaccination status. Most studies investigating in-hospital mortality by vaccination status provide unadjusted estimates with varying results from beneficial [8–14], through neutral [15–18] to harmful [19, 20] effects of vaccination. Given the fact that most countries prioritised high-risk patients (the elderly, those with comorbidities) for vaccination [21], these unadjusted estimates may reflect local factors and are less helpful for risk stratification of admitted patients. This is supported by the fact that the effect of vaccination on mortality changed from a harmful to a neutral [19] and from neutral to beneficial [18] after adjustment for age, sex, race, and comorbidities. Most studies that have taken into account determinants of vaccination found decreased mortality among vaccinated hospitalised patients compared to non-vaccinated patients [15, 22–25]; however, a large cohort of almost 3 thousand people reported null findings on both in-hospital and intensive care mortality [19].

Mortality estimates adjusted for the above variables may still be biased if vaccinated and unvaccinated patients are admitted at different level of disease severity. For example, it is conceivable that physicians unconsciously admit vaccinated patients with more severe presentation compared to unvaccinated patients given the striking effectiveness of COVID-19 vaccines. However, further adjustment for disease severity is rarely found in the literature. Given these, we aimed to investigate (1) univariate and independent determinants of COVID-19 vaccination, as well as (2) the effect of vaccination status on all-cause 30-day or in-hospital mortality in hierarchical models adjusted sequentially for determinants of vaccination status, laboratory markers of disease severity, and clinical predictors of mortality in a cohort of patients admitted for COVID-19 to a single tertiary hospital in Hungary during the pandemic wave dominated by the delta (B.1.617.2) variant.

## Materials and methods

### Setting

All adult patients were eligible to participate in this retrospective cohort study if they were admitted to the Department of Internal Medicine and Oncology, Semmelweis University, Budapest, Hungary, with a confirmed SARS-CoV-2 infection (based on real-time polymerase chain reaction or direct antigen tests) between 01/OCT/2021 and 15/DEC/2021. This time period completely overlaps with the period when > 98% of the sequenced variants were delta variants (B.1.167) [26].

The national vaccination programme started with the vaccination of healthcare workers and inhabitants of long-term care facilities in Hungary in the end of 2020. This was followed by vaccination of high-risk groups (the elderly and people with chronic medical conditions) and finally vaccination of the whole adult population started at the end of April 2021. The use of booster vaccines started in August 2021. Until the end of the study period, altogether 7 COVID-19 vaccines were used in Hungary: 2 mRNA-based (Comirnaty, Pfizer-BioNTech; Spikevax, Moderna), 3 viral vector-based (Vaxzevria, Astra-Zeneca; Gam-COVID-Vac, Gamaleya Research Institute of Epidemiology and Microbiology, and JCOVDEN, Janssen-Cilag), and an inactivated whole virus vaccine (BBIBP-CorV, Sinopharm) [27].

Baseline assessments including demography, medical history, vaccination status, laboratory findings, imaging results, and symptomatic and causal treatments were driven by institutional protocols. In brief, all patients admitted received low-dose low-molecular weight heparin (4000––6000 U/day sc.), oral dexamethasone (4–8 mg/day, a maximum for 10 days), and cholecalciferol (12,000 IU for 5 days and 3000 IU thereafter). The use of antiviral treatment was based on the degree of pulmonary involvement (based on chest X-ray or CT scans): no specific antiviral treatment for mild cases (< 25% of lung parenchymal involvement and no oxygen requirement), intravenous remdesivir for more severe cases (≥ 25% lung parenchymal involvement and/or requiring oxygen supplementation) for 5–10 days. In addition, reconvalescent plasma therapy, baricitinib, or tocilizumab were used in severe disease on a case-by-case basis [28, 29].

As no specific study-related procedures or data collections were performed in addition to routine care processes, no individual consent was sought for this retrospective analysis. Ethical approval was obtained from the Regional and Institutional Committee of Science and Research Ethics of Semmelweis University (RKEB 245/2020).

### Participants

**Fig. 1:**
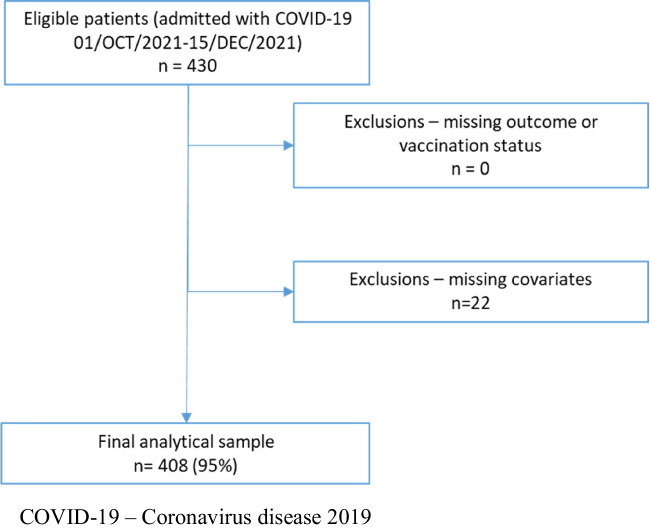
Flow chart of patient selection. COVID-19, coronavirus disease 2019

Altogether *n* = 430 patients were admitted to the Department in the study period. We had complete data on vaccination status and in-hospital mortality; however, we had to exclude *n* = 22 patients with missing covariates leading to a final analytical sample of *n* = 408 (Fig. 1).

### Outcome

All-cause mortality was drawn from electronic health records. Follow-up for living status started on the day of admission and participants were followed for 30 days or end of continuous hospitalisation whichever came later.

### Predictors and covariants

Vaccination status was coded as no or incomplete primary vaccination, full primary vaccination, or full primary plus booster vaccination. Full primary vaccination was defined according to the marketing authorisation for each vaccine (1 shot for the JCOVDEN and 2 shots for all other vaccines). Booster vaccination was defined as any vaccination following the complete primary vaccination. Although we collected data on the type of vaccines, given the limited number of participants, we did not analyse the type, the sequence, or the timing of the vaccines separately.

Among demographic characteristics, we included patient age and sex as potential covariates. The following known diseases in the medical history were collected and used in the analysis based on the literature: presence of hypertension, diabetes mellitus (including both type 1 and 2 diabetes), hyperlipidaemia, chronic obstructive pulmonary disease (COPD, any stage), atrial fibrillation (AF), chronic kidney disease (CKD, defined as eGFR < 60 ml/min), dementia, as well as prior cardiovascular disease (including history of stroke, myocardial infarction, peripheral artery disease, or hospitalisation due to heart failure), active treatment for or actual presence of malignancies or past history of malignancies without known activity [30–32].

All laboratory analyses were performed at the same central laboratory (Department of Laboratory Medicine of Semmelweis University) on automated systems at the time of admission. For the current analysis, we selected white blood cell count (WBC), percentage of neutrophils and lymphocytes, the level of C-reactive protein (CRP), and procalcitonin as markers of disease severity, while serum creatinine and serum albumin were used as markers of general health [32].

Pulmonary involvement was defined as any infiltrate on chest X-ray or CT scan reports. Although CT scans were routinely evaluated for the percentage of lung area involved in inflammation and CORADS score were also given, due to the high percentage of patients with only chest X-rays, we decided to use only a crude measure of pulmonary involvement.

### Statistical analysis

Descriptive statistics are provided stratified by primary vaccination status (full primary vaccination yes/no) and by survival status. Categorical variables are reported as numbers (percentages), and continuous variables as means ± standard deviations. Between group differences were calculated with chi-squared tests and independent sample *t*-tests as appropriate.

First, we investigated independent predictors of primary vaccination, as these variables are expected to confound the association between vaccination status and mortality. For this analysis, we entered in addition to age and sex, all variables from the medical history, and laboratory markers of general health that showed a univariate association with vaccination status (*p* < 0.10) into a logistic regression model with vaccination status as the outcomes using a backward stepwise method.

Then, we run a hierarchical logistic regression model with survival status as the outcome. We entered all independent predictors of primary vaccination into *model 1*, then we added the full vaccination status (no or partial — 0, full primary — 1, booster — 2, contrast: polynomial; *model 2*), then using backward stepwise entry, we added laboratory measures that could signal COVID-19 severity and were univariately associated with mortality (*model 3*), and finally variables from the medical history that were univariately associated with mortality (*model 4*). Results of the logistic regression models are given as odds ratios (OR) and 95% confidence intervals (CI) for 1 unit change for continuous variables and for the presence vs absence of any given dichotomous variables. We hypothesised that the 3 levels of full vaccination status were equally spaced and tested for the effect of it using a linear (polynomial) contrast.

As a sensitivity analysis, we investigated whether the effect of vaccination was similar in different age groups. Given the limited statistical power for this analysis, we used a dichotomous variable for vaccination (full or booster vs no or partial) and 3 age groups (< 70, 70–79.9, ≥ 80 years) with similar number of events.

IBM SPSS Statistics for Windows Version 28.0.1.0 (IBM Corporation, Armonk, NY, USA) was applied for all statistical analyses. A 2-sided *p* value < 0.05 was considered statistically significant. No adjustment for multiple tests was done, and all analyses were considered as hypothesis generating only.

## Results

### Patient characteristics by primary vaccination status

Vaccinated patients were (as expected) significantly older and had more frequently hypertension, diabetes mellitus, COPD, CKD, and malignancy (both active and past) in their medical history (all *p* < 0.05). The prevalence of other important comorbidities (hyperlipidaemia, myocardial infarction or heart failure, stroke, atrial fibrillation, dementia) was similar in the vaccinated and unvaccinated groups (all *p* > 0.05) (Table 1).

**Table 1:** Patient characteristics by primary vaccination status

Variable	Unvaccinated	Vaccinated	*p*
*n*	193	215	
Age (years)	62.5 ± 16	70.2 ± 14	< 0.0001
Male	107 (55.4%)	120 (55.8%)	1.00
Medical history			
Hypertension	114 (59.1%)	177 (82.3%)	< 0.0001
Diabetes mellitus	48 (24.9%)	81 (37.7%)	0.006
Hyperlipidaemia	52 (26.9%)	76 (35.3%)	0.07
Myocardial infarction/heart failure	32 (16.6%)	38 (17.7%)	0.794
Stroke	16 (8.3%)	18 (8.4%)	1.00
Chronic obstructive pulmonary disease	19 (9.8%)	36 (16.7%)	0.043
Atrial fibrillation	19 (9.8%)	33 (15.3%)	0.104
Chronic kidney disease	26 (13.5%)	56 (26%)	0.002
Dementia	11 (5.7%)	19 (8.8%)	0.258
Past history of malignancy	4 (2.1%)	18 (8.4%)	0.007
Currently active malignancy	16 (8.3%)	45 (20.9%)	< 0.0001
Measures at admission			
Pulmonary involvement	170 (88.1%)	163 (75.8%)	0.002
White blood cell count (G/l)	8.9 ± 8.8	9.7 ± 8.2	0.343
Neutrophils (%)	76.1 ± 12.6	77.4 ± 13.9	0.329
Lymphocytes (%)	16.2 ± 11.4	14.5 ± 10.6	0.117
Procalcitonin (ng/ml)	1.4 ± 8.3	1.6 ± 7.2	0.75
C-reactive protein (mg/l)	93.5 ± 79.4	102.2 ± 77.8	0.267
Serum creatinine (µmol/l)	132.3 ± 156	157 ± 162.3	0.12
Serum albumin (g/l)	32 ± 5	30.8 ± 5.6	0.04
Outcome			
Death	50 (25.9%)	60 (27.9%)	0.657

Mean ± SD or *n* (%) as appropriate

*p* values are given for independent sample *t*-tests for continuous and for chi-squared tests for categorical variables

In addition, vaccinated patients presented more frequently with pulmonary involvement and had lower serum albumin at the time of admission (all *p* < 0.05). However, vaccinated and unvaccinated patients presented with similar inflammatory measures (WBC, percentage of neutrophils and lymphocytes, procalcitonin, and CRP) and serum creatinine levels (all *p* > 0.05). Finally, unadjusted mortality was similar (~ 25–30%) among both vaccinated and unvaccinated patients (Table 1).

### Independent predictors of vaccination

Next, we built a multiple logistic regression model with primary vaccination status as the outcome and age, hypertension, CKD, and malignancy in the medical history, as well as serum albumin at hospital admission as potential covariates. After backward elimination, the final model included older age, presence of hypertension, CKD, and both active and past malignancy as independent predictors of primary vaccination (Table 2).

**Table 2:** Independent predictors of primary vaccination based on multiple logistic regression

Variable	Odds ratio	95% confidence interval
Age	1.02	1.01–1.04
Hypertension	2.05	1.18–3.55
Chronic kidney disease	1.78	0.99–3.2
Currently active malignancy	4.31	1.22–15.24
Past history of malignancy	2.77	1.44–5.32

Other variables available for the model: serum albumin at hospital admission

### Patient characteristics by survival status

Deceased patients were significantly older; had more frequently hypertension, myocardial infarction or heart failure, atrial fibrillation, CKD, and active malignancy in their medical history; had more pronounced markers of inflammation (i.e. had higher neutrophil and lower lymphocyte relative counts, higher CRP and procalcintonin levels); and higher serum creatinine and lower albumin level at the time of admission (all *p* < 0.05). The prevalence of hyperlipidaemia, prior stroke, and dementia also was higher among deceased people as well as WBC, although these differences did not reach statistical significance (all *p* < 0.1) (Table 3).

**Table 3:** Patient characteristics by survival status

Variables	Alive	Deceased	*p*
n	298	110	
Age (years)	63.5 ± 15.6	74.7 ± 11.4	< 0.0001
Male	171 (57.4%)	56 (50.9%)	0.262
Medical history			
Hypertension	199 (66.8%)	92 (83.6%)	< 0.0001
Diabetes mellitus	86 (28.9%)	43 (39.1%)	0.055
Hyperlipidaemia	86 (28.9%)	42 (38.2%)	0.092
Myocardial infarction/heart failure	44 (14.8%)	26 (23.6%)	0.039
Stroke	20 (6.7%)	14 (12.7%)	0.068
Chronic obstructive pulmonary disease	37 (12.4%)	18 (16.4%)	0.328
Atrial fibrillation	28 (9.4%)	24 (21.8%)	0.001
Chronic kidney disease	44 (14.8%)	38 (34.5%)	< 0.0001
Dementia	17 (5.7%)	13 (11.8%)	0.052
Past history of malignancy	16 (5.4%)	6 (5.5%)	1.00
Currently active malignancy	34 (11.4%)	27 (24.5%)	0.002
Measures at admission			
Pulmonary involvement	238 (79.9%)	95 (86.4%)	0.11
White blood cell count (G/l)	8.8 ± 7.5	10.8 ± 10.5	0.062
Neutrophils (%)	75.2 ± 12.6	81.2 ± 14.4	< 0.0001
Lymphocytes (%)	16.7 ± 10.8	11.6 ± 10.9	< 0.0001
Procalcitonin (ng/ml)	0.9 ± 5.6	3.3 ± 11.6	0.035
C-reactive protein (mg/l)	86.6 ± 71	129.2 ± 89.4	< 0.0001
Serum creatinine (µmol/l)	129.6 ± 147.8	187.9 ± 181.9	0.003
Serum albumin (g/l)	32.2 ± 4.9	28.9 ± 5.8	< 0.0001
Vaccination status			0.712
Unvaccinated/partially vaccinated	134 (45%)	46 (41.8%)	
Full primary vaccination	122 (40.9%)	50 (45.5%)	
Booster vaccinated	42 (14.1%)	14 (12.7%)	
Vaccinated	155 (52%)	60 (54.5%)	0.657

Mean ± SD or *n* (%) as appropriate

*p* values are given for independent sample *t*-tests for continuous and for chi-squared tests for categorical variables

We found no difference in the sex distribution, in the prevalence of COPD, medical history of past malignancy, the presence of pulmonary involvement at admission, or the vaccination status between deceased and surviving patients (Table 3).

### Independent predictors of 30-day all-cause mortality

According to our final model, the independent predictors of all-cause mortality were older age, the presence of chronic kidney disease, currently active malignancy, and atrial fibrillation, lower percentage of lymphocytes, and higher C-reactive protein levels (Table 4).

**Table 4:** Independent predictors of 30-day all-cause mortality based on hierarchical multiple logistic regression

Variables	Model 1			Model 2			Model 3			Model 4		
	Odds ratio	95% CI	*p*	Odds ratio	95% CI	*p*	Odds ratio	95% CI	*p*	Odds ratio	95% CI	*p*
Age (years)	1.06	1.04–1.08	< 0.001	1.06	1.04–1.08	< 0.001	1.06	1.04–1.09	< 0.001	1.06	1.04–1.09	< 0.001
Hypertension	1.04	0.53–2.02	0.912	1.18	0.6–2.32	0.629	1.04	0.51–2.1	0.922	0.96	0.47–1.97	0.915
Chronic kidney disease	2.73	1.57–4.77	< 0.001	2.96	1.67–5.25	< 0.001	2.96	1.62–5.4	< 0.001	2.97	1.63–5.44	< 0.001
Currently active malignancy	2.28	1.24–4.2	0.008	2.62	1.39–4.92	0.003	2.59	1.33–5.03	0.005	2.64	1.35–5.13	0.004
Past history of malignancy	0.76	0.27–2.12	0.595	0.72	0.25–2.08	0.545	0.62	0.2–1.88	0.393	0.63	0.21–1.92	0.416
Vaccination status						0.057			0.027			0.024
Linear				0.51	0.29–0.89	0.017	0.45	0.25–0.81	0.007	0.44	0.24–0.79	0.006
Deviation				0.89	0.58–1.37	0.589	0.83	0.52–1.3	0.412	0.84	0.53–1.32	0.441
Lymphocytes (%)							0.97	0.94–1	0.036	0.97	0.94–1	0.042
C-reactive protein (mg/l)							1.01	1–1.01	< 0.001	1.01	1–1.01	< 0.001
Atrial fibrillation										1.86	0.93–3.71	0.078

*95% CI*, 95% confidence interval

*Model 1*, independent predictors of vaccination (age, hypertension, chronic kidney disease, currently active malignancy, past history of malignancy; method: enter)

*Model 2*, model 1 + vaccination status (no or partial — 0, full primary — 1, booster — 2; method: enter, contrast: polynomial)

*Model 3*, model 2 + laboratory measures (white blood cell count, neutrophils, lymphocytes, procalcitonin, C-reactive protein, serum creatinine, serum albumin; method: backward stepwise)

*Model 4*, model 3 + medical history (diabetes mellitus, hyperlipidaemia, myocardial infarction/heart failure, stroke, atrial fibrillation, dementia; method: backward stepwise)

**Fig. 2:**
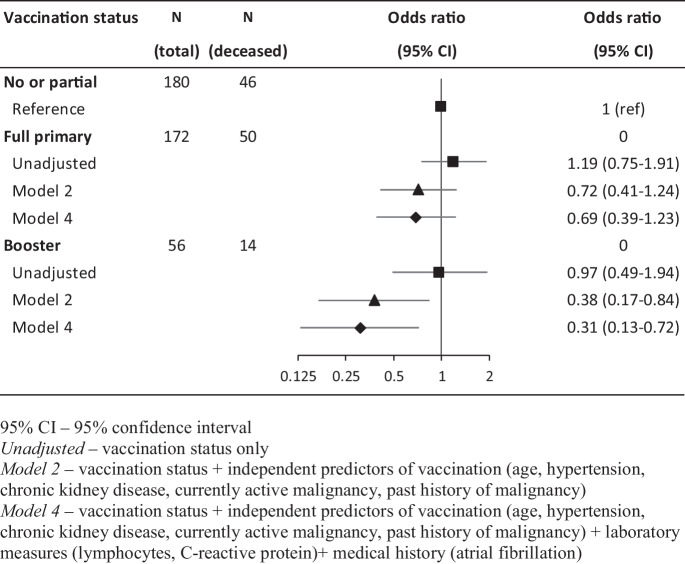
Association between vaccination status and 30-day all-cause mortality

Regarding the role of vaccination status, we found a linear decrease in mortality from no/partial primary vaccination through full primary vaccination to booster vaccination that translates to a non-significant, approximately 30% reduction in risk in patients with full primary vaccination and a 60–70% risk reduction in booster-vaccinated patients. The importance of the different levels of adjustment is supported by the fact that while there was no association between vaccination status and mortality in unadjusted models, sequential adjustment for predictors of vaccination, laboratory measures of severity, and comorbid conditions strengthened the association (Table 4, Fig. 2).

### Sensitivity analysis

Our sensitivity analysis investigating the potential effect modification by age (although had limited statistical power) showed similar point estimates within the 3 age groups and to that of the main analysis with completely overlapping confidence intervals (Supplementary Table 1).

## Discussion

According to our results on a cohort of sequentially admitted patients with COVID-19 during the delta wave of the pandemic in Hungary, we found that vaccination status was an important predictor of all-cause 30-day mortality when we adjusted for determinants of vaccination, disease severity, and comorbid conditions. While we found a non-significant (~ 30%) risk reduction in patients with full primary vaccination, patients that also received a booster vaccination had an ~ 70% risk reduction. Overall, there was a significant linear trend along the level of vaccination among hospitalised patients. Other important predictors of all-cause mortality were older age, the presence of chronic kidney disease, currently active malignancy, and atrial fibrillation, lower percentage of lymphocytes, and higher C-reactive protein levels.

### Importance of the research question and potential hurdles of its investigation

There are several aspects related to the efficacy and the effectiveness of COVID-19 vaccination in relation to different outcomes and variants of SARS-CoV-2 that require further clarification. The effectiveness of a full primary vaccination against SARS-CoV-2 in terms of symptomatic COVID-19 has been unambiguously proven both in terms of the alpha and the delta variants in the general population [33]. Furthermore, these vaccines retain their effectiveness against hospitalisations for at least 6 months. [34] Although some waning of vaccine effectiveness is evident dependent on age, different virus variants, vaccine type, and the outcome [35], their effectiveness lasts for at least 4 months. [36] It was also clearly shown that the mRNA-based vaccines in addition to effectively reducing hospitalisations have a marked effect on disease progression to death or the necessity of mechanical ventilation compared to unvaccinated patients [24].

While the above randomised trials and real-word studies performed in the general population give crucial evidence for the planning of vaccination programmes against COVID-19 and for the distribution of healthcare resources, they are unable to answer a crucial question for the patient and healthcare provider, whether patients requiring hospitalisation have different outcomes by vaccination status. Given that vaccination effectively reduces both hospitalisations and all-cause mortality, its effect on mortality after hospitalisation is hard to predict. Furthermore, the investigation of this question is hindered by several potential drawbacks that should be considered. First, vaccinated people are very different from non-vaccinated people, as vaccination is targeted to high-risk patients (elderly, those with comorbidities, etc.). Second, disease severity could be different between vaccinated and unvaccinated patients at hospital admission due to selection bias requiring adjustment. Furthermore, in multicentre studies, local capacity and protocols could lead to different populations in each centre. However, single-centre investigations (given the lower number of patients) will have limited power to adjust for confounders.

### Unadjusted studies

As expected, the crude effect of vaccination on mortality of patients hospitalised with COVID-19 shows a wide variation. While most studies show a lower mortality among vaccinated patients [8–14], there are at least 4 studies that show neutral association [15–18], while two studies report higher mortality among vaccinated hospitalised patients compared to unvaccinated individuals [19, 20]. While some of these studies included patients infected with other than the delta variant of SARS-CoV-2, the vector does not seem to be related to this outcome as studies restricted to the delta variant [10, 13, 15, 16, 18], as well as those including a mixture [8, 9, 17], show both beneficial and neutral effects of vaccination on mortality. While it seems plausible that the neutral effects could be related to low number of participants, most of these studies were regional or multicentre investigations [15, 16], and one was a meta-analysis [17] that argues against a lack of sufficient power. We suspect that indications for vaccination in the national programmes (older age, higher number of certain comorbidities) created baseline differences in these risk factors of COVID-19-related mortality favouring unvaccinated people as it was also found in our analysis. It is interesting to note that none of the studies investigating patients hospitalised in intensive care units (ICU) showed better survival in vaccinated patients [16, 19]. This observation might suggest that vaccinated hospitalised patients were less likely to require (a potential consequence of vaccination) or receive (a potential consequence of unconscious bias) ICU care. Our findings show no crude effect of vaccination on mortality although with wide confidence intervals. Interestingly, a population-based study from Hungary that includes all COVID-19 cases 65 years or older showed lower mortality among vaccinated patients with a 30–40% relative risk reduction with full or booster vaccination [12]. These results may point toward a larger effect on mortality in the elderly, or may argue that older age is the one, most important baseline difference between vaccinated and unvaccinated patients at hospital admission.

These unadjusted comparisons should be interpreted with utmost care, as these studies were completed in different countries with fairly different healthcare systems (e.g. the available nurse care and the capacities are completely different).

### Studies with different level of adjustment

In contrast to unadjusted estimates, the risk of mortality adjusted for different confounders shows a much clearer picture. Most evidence supports a protective effect associated with vaccination [12, 15, 18, 22, 24, 25], while two reports show neutral effects [19, 37]. One of the neutral observation is a case–control study, where matching was based on age and sex. However, this seems to be insufficient level of adjustment, as vaccinated people are not only older but have more comorbidities as shown by our results [37]. The other was a multicentre study conducted in ICUs. We suspect that admission to ICUs requires such a risk that is equalising mortality risk independent of vaccination status [19].

Most studies adjusted for the presence, number of, or individual comorbidities in addition to age, sex, and ethnicity. These studies (similarly to our one) report stronger protective effect of vaccination on all-cause mortality compared to unadjusted analyses showing 50 to 90% lower odds of death with full vaccination in adjusted models [12, 15, 18, 22, 24, 25]. These observations well correspond to our finding of a 30–70% reduced risk of mortality associated with a full and a booster vaccination. It is interesting to note that the protective effect of vaccination seems to be much stronger (70 and 80% for full and booster vaccination) than that reported in the present paper in an analysis that included all Hungarian patients over 65 years of age hospitalised during the delta wave [12].

While it is well accepted that in addition to comorbidities, several biological measures at admission are important predictors of mortality and ICU admission [38–40], they were rarely included as co-variables in the multiple adjusted models [18]. Our study benefited from the availability of a wide range of severity measures at admission.

### Strengths and limitations

Our study has certain strengths that should be highlighted. As our hospital served as dedicated hospital for the care of COVID-19 patients in a certain geographical area, our population-based results probably have a good external validity to similar institutions. Furthermore, care and treatment were driven by standardised protocols that explain the low proportion of missing data and provide good internal validity to our findings. Similarly, we had no missing data on 30-day mortality due to the use of nationwide health records. Furthermore, all imaging and laboratory measurements were performed in the same institution with appropriate quality control. The detailed phenotyping of the patients allowed us to adjust for the most important co-variables including disease severity measures. It is notable that we had a framework for the adjustment of different co-variables including determinants of vaccination and survival of COVID-19 patients. Another important aspect of our study is the potential for investigating the dose–response effect of full and booster vaccination status on survival.

However, some limitations should be acknowledged. The potential role of selection bias cannot be downplayed. Selection bias both related to admissions and to transfers to intensive care could limit the external validity of our findings. As far as admissions are concerned, the Hungarian healthcare system was by and large able to cope with the number COVID-19 patients although it is possible that some of the mild cases that would have been hospitalised otherwise were deferred. Similarly, the intensive care unit at our hospital system was also able to cope with the number of patients requiring further treatment. It should be noted that departments without experience in infection diseases (such as urology) were involved in the care of COVID-19 patients during the whole pandemic and thus theoretically it is possible that triage directed more severe cases to our hospital. Overall, we think that our results have a good external validity for healthcare systems in high-income countries with sufficient resources to provide necessary care for most patients.

Given the single-centre nature of our investigation, the number of participants is relatively low that limits statistical power. Indeed, we think that the non-significant protective effect of a full vaccination reflects this power issue. The limited power precluded the comparison of the used vaccines or the investigation of the modifying effect of time since the last vaccination on mortality. We limited our analysis to the investigation of mortality during the delta wave. While this further limited the number of participants, this way we could remove the different effect of different virus variants on mortality [41]. To overcome this limitation of the present study, we plan to extend data collection to other waves of the pandemic in the same department, as well as to include patients from other departments at the same healthcare system that used the same treatment protocols.

It should be noted that we had no data on some important potential confounders (such as detailed description of CT scans) and thus our estimates on protection are probably conservative. Furthermore, although there are over a hundred risk factors of COVID-19-related mortality reported in a systematic review but our set of variables covered most preexisting conditions and types of laboratory parameters (i.e. inflammation, haemostasis) identified [32].

## Conclusions

Our study clearly showed a dose–response between vaccination status and 30-day all-cause mortality when important predictors of vaccination and COVID-19-related mortality were taken into account. These findings highlight the fact the protective effect of vaccination extends to those people requiring hospitalisation due to COVID-19. Furthermore, our results clearly support the hypothesis that booster vaccinations further improve protection of hospitalised patients, although the role of the time gap between vaccination and disease onset requires further clarification. We think that this and similar studies in future pandemics could help improving the patient-provider discussions on perceived risk of mortality at the time of admission.

### Supplementary Information

Below is the link to the electronic supplementary material.

Supplementary file1 (DOCX 19 KB)

## Data Availability

NA.
