# Supplementary material for: The effect of COVID-19 vaccination status on all-cause mortality in patients hospitalised with COVID-19 in Hungary during the delta wave of the pandemic
Source: GeroScience. 2023 Sep 27;46(2):1881–94. doi: 10.1007/s11357-023-00931-1 (PMC10828407; doi:10.1007/s11357-023-00931-1)
Supplement: Supplementary file 1 — Supplementary file1 (DOCX 19 KB) [file 11357_2023_931_MOESM1_ESM.docx]

# Supplementary material

# The effect of COVID-19 vaccination status on all-cause mortality in patients hospitalized with COVID-19 in Hungary during the delta wave of the pandemic

**Viktor J Horvath, MD, PhD^1^*, Magdolna Békeffy, MD^1^*, Zsuzsanna Németh, PhD^1^, Emese Szelke, MD, PhD^1^, Vince Fazekas-Pongor, MD, PhD^3^, Noémi Hajdu, MD^1^, Márk M Svébis, MD^1^, József Pintér, MD^1^, Beatrix A Domján, MD^1^, Szilvia Mészáros, MD, PhD^1^, Anna E Körei, MD, PhD^1^, Árpád Kézdi, Drd^1^, Ibolya Kocsis, MD, PhD^2^, Katalin Kristóf, MD, PhD^2^, Prof Péter Kempler, MD, PhD, DSc^1^, Prof Ferenc Rozgonyi, MD, PhD, DSc^2^, Prof István Takács, MD, PhD, DSc^1^, Prof Adam G Tabák, MD, PhD^1,3,4^**

**^1^** Department of Internal Medicine and Oncology, Semmelweis University Faculty of Medicine, Budapest, Hungary

^2^ Department of Laboratory Medicine, Semmelweis University Faculty of Medicine, Budapest, Hungary

**^3^** Department of Public Health, Semmelweis University Faculty of Medicine, Budapest, Hungary

**^4^** UCL Brain Sciences, University College London, London, UK

* Authors contributed equally

Corresponding author: Viktor J Horváth, Department of Internal Medicine and Oncology, Semmelweis University Faculty of Medicine, 2/a Korányi S. Str., H-1083 Budapest, Hungary

e-mail: [horvathjviktor@gmail.com](mailto:horvathjviktor@gmail.com)

**Supplementary Table 1** – Independent predictors of 30-day all-cause mortality based on hierarchical multiple logistic regression

| **Variables** | **Model 1** | | | **Model 2** | | | **Model 3** | | | **Model 4** | | |
| --- | --- | --- | --- | --- | --- | --- | --- | --- | --- | --- | --- | --- |
|  | **odds ratio** | **95% CI** | **p** | **odds ratio** | **95% CI** | **p** | **odds ratio** | **95% CI** | **p** | **odds ratio** | **95% CI** | **p** |
| ***Age (years)*** | 1.06 | 1.04-1.08 | <0.001 | 1.06 | 1.03-1.08 | <0.001 | 1.06 | 1.04-1.09 | <0.001 | 1.06 | 1.04-1.09 | <0.001 |
| ***Hypertension*** | 1.04 | 0.53-2.02 | 0.912 | 1.16 | 0.59-2.27 | 0.679 | 1.02 | 0.51-2.03 | 0.962 | 0.94 | 0.47-1.89 | 0.867 |
| ***Chronic kidney disease*** | 2.73 | 1.57-4.77 | <0.001 | 2.96 | 1.67-5.25 | <0.001 | 2.91 | 1.58-5.36 | <0.001 | 2.93 | 1.60-5.37 | <0.001 |
| ***Currently active malignancy*** | 2.28 | 1.24-4.2 | 0.008 | 2.66 | 1.41-5.02 | 0.003 | 2.67 | 1.38-5.16 | 0.004 | 2.71 | 1.40-5.22 | 0.003 |
| ***Past history of malignancy*** | 0.76 | 0.27-2.12 | 0.595 | 0.86 | 0.30-2.46 | 0.782 | 0.78 | 0.3-2.01 | 0.626 | 0.80 | 0.30-2.12 | 0.660 |
| ***Vaccinated by Age group1*** |  |  |  | 0.50 | 0.23-1.05 | 0.068 | 0.49 | 0.23-1.05 | 0.065 | 0.50 | 0.23-1.07 | 0.073 |
| ***Vaccinated by Age group2*** |  |  |  | 0.60 | 0.31-1.17 | 0.134 | 0.51 | 0.25-1.03 | 0.060 | 0.49 | 0.24-0.99 | 0.047 |
| ***Vaccinated by Age group3*** |  |  |  | 0.56 | 0.25-1.29 | 0.176 | 0.53 | 0.22-1.30 | 0.166 | 0.52 | 0.21-1.31 | 0.164 |
| ***Lymphocytes (%)*** |  |  |  |  |  |  | 0.97 | 0.94-1.00 | 0.034 | 0.97 | 0.94-1.00 | 0.039 |
| ***C-reactive protein (mg/l)*** |  |  |  |  |  |  | 1.01 | 1.003-1.01 | <0.001 | 1.01 | 1.00-1.01 | <0.001 |
| ***Atrial fibrillation*** |  |  |  |  |  |  |  |  |  | 1.84 | 0.93-3.67 | 0.081 |

95% CI – 95% confidence interval

Age group1 – age <70 years

Age group2 – age 70 to <80 years

Age group3 – age ≥80 years

Vaccinated – vaccination status (no or partial vs full primary or booster

*Model 1* – independent predictors of vaccination (age, hypertension, chronic kidney disease, currently active malignancy, past history of malignancy; method: enter)

*Model 2* – Model 1 + vaccination status (no or partial vs full primary or booster; method: enter)

*Model 3* – Model 2 + laboratory measures (white blood cell count, neutrophils, lymphocytes, procalcitonin, C-reactive protein, serum creatinine, serum albumin; method: backward stepwise)

*Model 4* – Model 3 + medical history (diabetes mellitus, hyperlipidaemia, myocardial infarction/heart failure, stroke, atrial fibrillation, dementia; method: backward stepwise)
